# Remission from depression is associated with improved quality of life and preserved exercise capacity in adults with congenital heart disease

**DOI:** 10.3389/fcvm.2024.1418342

**Published:** 2024-07-03

**Authors:** Brit Fillies, Britta Stapel, Lars H. Lemke, Friederike Löffler, Johann Bauersachs, Kai G. Kahl, Mechthild Westhoff-Bleck

**Affiliations:** ^1^ Department of Cardiology and Angiology, Hannover Medical School, Hanover, Germany; ^2^ Department of Psychiatry, Social Psychiatry and Psychotherapy, Hannover Medical School, Hanover, Germany

**Keywords:** depression, remission from depression, quality of life, cardiorespiratory function, exercise capacity, adult congenital heart disease

## Abstract

**Aims:**

Improved long-term survival has widened the treatment goals for adults with congenital heart disease (ACHD) by addressing parameters that impact mental well-being and exercise capacity. Depression, a frequent co-morbidity in ACHD, is linked to both. Whether successful treatment of depression also affects cardiac parameters is a matter of debate.

**Methods:**

This prospective, cross-sectional, longitudinal study included *N* = 150 ACHD (mean age 35.2 ± 11.3 years, 57% male) at baseline (t0) and *N* = 114 at follow-up (mean follow-up: 4.8 ± 0.6 years; t1). Patients were interviewed using a structured clinical interview, and severity of depression was assessed using the Montgomery-Asperg Depression Scale (MADRS). Additional testing was performed using self-rating questionnaires concerning depression, anxiety and quality of life (QoL). Exercise capacity (VO_2max_) was assessed by symptom limited exercise testing.

**Results:**

Of *N* = 33 patients diagnosed with depression at t0, *N* = 18 patients remitted and *N* = 15 were non-remitters. Remitters displayed significantly decreased anxiety (*P *= 0.013), improved global QoL (*P *= 0.002), and preserved VO_2max_ (*P *= 0.958) at t1 compared to t0. This was associated with favourable health behaviour at t1 and stable body-mass-index. Contrarily, non-remitters reported further increased anxiety (*P *= 0.021) and no significant improvement in QoL (*P *= 0.405). VO_2max_ declined significantly (*P *= 0.006) and body-mass-index increased (*P *= 0.004). Never-depressed patients showed no significant changes in anxiety (*P *= 0.415) or QoL (*P *= 0.211). VO_2max_ decreased significantly (*P *< 0.001).

**Conclusion:**

In ACHD, remission from depression is associated with better physical functioning, mental health, and QoL. The assessment and treatment of depression in ACHD emerges as an important clinical goal that should be included in a comprehensive multimodal treatment plan.

## 1 Introduction

Congenital heart disease (CHD) constitutes the most common single birth defect; it affects approximately 1% of all live birth (1, 2). Owing to medical advances, in industrialized countries more than 95% of children with CHD now reach adulthood, leading to a continuously growing patient population of adults with a great heterogeneity of heart defects (ACHD) (3, 4). In this regard, the number of ACHD patients is estimated at 50 million worldwide with a steady increase projected in the next decades (5, 6). With the improvement in long-term survival, additional treatment goals have been become a more prominent focus in the care for ACHD patients. Accordingly, research in the cardiovascular field has expanded from studies focussing on survival and mortality to studies that include patient-reported outcomes. For instance, health-related quality of life (QoL) has become an important outcome measure in clinical research (7, 8). The importance of QoL expands beyond a prognostic marker as patients attribute a higher importance to it than to longevity (9, 10).

Next to patient-reported outcomes, surrogate measures for hard outcomes are commonly applied in the context of ACHD. Particularly, maintenance and improvement of exercise capacity presents an important treatment outcome as it has been shown to have a strong independent prognostic value in ACHD patients regarding morbidity and mortality (11–13). Accordingly, modifiable factors that adversely impact exercise capacity present an intriguing research focus in the context of ACHD. Exercise capacity is defined as the maximum sustainable amount of physical exertion (14). In this regard, lower peak oxygen consumption (VO_2max_) levels, which constitute a key measure of exercise capacity, have been reported (15, 16). Additionally, the age-related decline in exercise capacity appears to be accelerated in patients with ACHD and low levels of exercise capacity have been associated with poor QoL in this patient population (17). Several factors have been suggested to adversely impact exercise capacity in ACHD patients. These include residual electrophysiological and hemodynamic defects and impairment of other organs connected to the CHD (18, 19). Importantly, studies have shown that patients with ACHD often do not follow physical activity guidelines as recommended for the general population (20, 21). Therefore, a sedentary lifestyle and adverse health behaviours might contribute to lower exercise capacity in ACHD patients. Importantly, dedicated literature suggests that exercise capacity of ACHD patients can be improved by frequent physical exercise (22).

Psychiatric disorders, particularly depression that has been shown to affect more than one third of ACHD patients (23–25). Indeed, a recent study including a global sample of over 3,000 ACHD patients concluded that approximately one third had elevated depression and/or anxiety scores (26). Additionally, particularly depression might constitute a common link to adverse QoL as well as to decreased exercise capacity in this patient population. In this regard, the association of depression and depressive symptoms with low QoL is well established in ACHD (23, 26, 27). Additionally, reduced exercise capacity, age, and anatomical complexity of the underlying heart defect have been established as significant moderators of QoL in ACHD (25, 27, 28).

Data regarding the impact of depression on exercise capacity in ACHD patients are limited and studies exploring the effect of depression on exercise capacity over time are lacking. However, cross-sectional studies assessing exercise capacity in other cardiac patient populations, for instance in patients with heart failure (22) and stable coronary artery disease (29), reported an association of depressive symptoms with decreased exercise capacity. Finally, depression regardless of somatic comorbidity, is frequently associated with a sedentary lifestyle and adverse lifestyle behaviours, including increased nicotine and alcohol consumption, and increased calory intake, which might contribute to adverse effects on exercise capacity (30).

Taken together, the literature points towards a complex interplay between underlying congenital heart disease, exercise capacity, QoL, and depression. Nevertheless, the role of depression is sparsely examined in prospective studies in ACHD.

In the present study, we investigated changes in exercise capacity, mental well-being and QoL in patients with ACHD, depending on depression status at baseline compared to a five-year follow-up.

## 2 Methods

### 2.1 Study design and participants

The PsyConHeart study was approved by the Institutional Ethical Review Board at Hannover Medical School, Germany (No. 6455/2013). Initially, *N* = 150 patients with ACHD were recruited from the ACHD outpatient Clinic of Department of Cardiology and Angiology at Hannover Medical School. Extensive study details are given in Westhoff-Bleck et al. (23). Inclusion criteria were the diagnosis of a structural CHD, sufficient German language skills to read and understand the questionnaires, and an age older than 18 years. The following exclusion criteria applied: pregnancy and an instability of the cardiac condition.

Of the initially at baseline (t0) included *N* = 150 ACHD patients, *N* = 145 were successfully contacted at the 5-year follow-up time point (4.8 ± 0.6 years, t1.) Five patients died during the follow-up period, of whom *N* = 4 had initially been diagnosed with major depression. Data from *N* = 114 patients that agreed to participate in the follow-up (also see results 3.1) were included in the study sample. At baseline, the mean age of patients was 34.8 years [standard deviation (SD): ± 11.4 years; range: 18–70 years]. *N* = 62 (54%) of patients were male and *N* = 52 (47%) were female. All patients graduated from school and had sufficient German language skills to participate in the study. Additional baseline data regarding the severity of the cardiac condition as well as measures of mental well-being and health-related QoL of the study population are supplied in Supplementary Table S1. Data regarding the complexity (Bethesda class) and type (diagnosis) of the underlying congenital heart defect as well as pharmacological treatment of the cardiovascular condition at baseline are depicted for the complete sample in Supplementary Table S2.

### 2.2 Psychometric testing and lifestyle assessment at t0 and t1

Depression diagnosis was obtained by Structured Clinical Interview (SCID) for Diagnostic and Statistical Manual of Mental Disorders (DSM)-IV (31). The SCID represents the current gold-standard of psychiatric diagnostics. It is a semi-structured interview guide that was applied by experienced psychiatrists and psychologists. All patients that received a depression diagnosis at t0 were recommended guideline-based depression treatment.

Depressive symptoms were assessed by expert-based interview as well as by self-rating. For expert-rating, the Montgomery-Asperg Depression Rating Scale, MADRS) (32) was applied. The MADRS consists of ten items that relate to symptoms of depression, next to others including reported sadness, inner tension, reduced sleep and appetite, numbness, pessimistic thoughts and suicidal thoughts. These are rated on a seven-point scale according to reported symptom severity. The MADRS measures symptom severity within the last week.

For self-rating of depressive symptoms, the depression subscale of the Hospital Anxiety and Depression Scale (HADS-D) (33) was utilized. Additionally, symptoms of anxiety were rated by use of the anxiety subscale of the HADS (HADS-A) (33). The HADS constitutes a 14-items containing questionnaire, with seven items relating to depression and seven items relating to anxiety. Each item is scored on a 4-point scale according to experienced symptom severity. The HADS measures symptom severity within the last two weeks. Specific cut-off values have been established for patients with ACHD (34).

Health-related QoL was measured using the short form of the World Health Organization QoL score (WHOQOL-BREF) (35). The WHOQOL-BREF constitutes an established patient reported outcome instrument that measures the global health status of patients independent of specific diseases across four health domains, i.e., physical health, psychological health, social relationships, and environment. The questionnaire contains 26 items measured on a 5-point scale, and assesses the health-related QoL within the last two weeks.

Alcohol consumption was recorded as drinks/week, smoking status in binary form as existing/absent nicotine consumption. The sport score was recorded on a modified 6-point Likert scale defined as lacking sports, occasional physical activity, frequently going out for a walk/light physical activity <1 week, once/week moderate sports activity/frequent biking, >1/week moderate physical exertion or exercising >3 times/week (36).

### 2.3 Cardiologic assessment at t0 and t1

All patients were examined by a cardiologist specialized in ACHD. Routine cardiologic examination included medical history, including number of surgical procedures, laboratory data, i.e., N-terminal prohormone of brain natriuretic peptide (NT-proBNP) and high-sensitivity C-reactive protein (CRP), and echocardiography to assess left ventricular morphology and systolic as well as diastolic ventricular function. Functional status was classified according to the New-York Heart Association (NYHA) Classification of heart failure, in which NYHA class 1 indicates no limitation-, NYHA class 2 slight limitation-, and NYHA class 3 marked limitation of physical activity, while NYHA class 4 is defined as unable to carry out any physical activity without discomfort (37). The Bethesda classification was used to classify the complexity of the underlying CHD in simple, moderate, and great (38).

### 2.4 Cardiorespiratory exercise testing at t0 and t1

Symptom-limited exercise testing was performed on a bicycle in an upright position. The workload steadily increased by 25 Watt every 2 min. Cardiorespiratory exercise testing (CPET) allows measurement of ventilation and respiratory gas exchange parameters in relation to work performance. The CPET derived maximum oxygen uptake (VO_2max_) measures peak functional exercise performance, which represents a well-established objective dimension of maximum functional capacity allowing assessment of heart failure severity and prognosis of cardiac diseases.

### 2.5 Statistical analysis

This prospective cross-sectional longitudinal observational study evaluated the impact of depression on QoL, self-reported symptoms of depression and anxiety, health behaviour and cardiorespiratory function. Continuous variables are presented as mean plus standard deviation (SD), if not indicated otherwise. Categorical variables are depicted as percentages or absolute values. The study groups were defined as “never-depressed” [no depression diagnosis at baseline (t0) and follow-up (t1)], “non-remitters” (depression diagnosis at both analytical timepoints), and “remitters” (depression diagnosis at t0 and no depression diagnosis at t1). Group comparisons of nominal data were performed using chi-square test. For continuous data the effect of depression over time, was assessed by use of repeated measures analysis of variance (ANOVA) for each dependent variable as indicated in the results section. Group differences at individual time points and time-dependent effects within each study group were assessed by pairwise comparisons. Bonferroni correct *P*-values are depicted and *P* < 0.05 was considered statistically significant. All statistical analyses were performed using SPSS (Version 24).

## 3 Results

### 3.1 Factors affecting participation and remission rates

Overall, *N* = 114 (81%) of contacted ACHD patients agreed to participate in the follow-up assessment. Of the *N* = 27 (19%) patients who declined to participate, *N* = 3 (11%) patients had moved away, *N* = 15 (56%) respondents cited professional reasons, and *N* = 9 (33%) cited private reasons. Non-participation at t1 was independent from diagnosis of depression at t0, (data not shown).

Of the *N* = 114 that agreed to participate in the follow-up survey, *N* = 81 (71%) patients did not meet the criteria for major depression at both t0 and t1 (never-depressed). The remaining *N* = 33 (29%) patients were initially diagnosed with major depression. Of the patients that received a depression diagnosis at t0, *N* = 15 (45%) patients had not remitted from depression (non-remitters), and *N* = 18 (55%) presented with remission from depression (remitters) at follow-up. Four patients received a new diagnosis of depression at t1. This group was withdrawn from further analysis. Supplementary Figure S1 provides an overview regarding group affiliation of ACHD patients.

Groups based on depression status did not significantly differ with regard to complexity (Bethesda class) of the underlying congenital heart defect. Further, with the exception of the diagnosis of “D-transposition: atrial switch”, no significant differences regarding type of the underlying congenital heart defect were observed (Supplementary Table S3) and pharmacological treatment of present cardiovascular disease did not significantly differ between groups at baseline (Supplementary Table S4).

Finally, of all patients diagnosed with depression at t0, *N* = 17 (52%) did not receive treatment for depression, while *N* = 3 (10%) received psychopharmacotherapy, *N* = 8 (24%) received psychotherapy and *N* = 5 (15%) received both. Both depression groups, i.e., remitter and non-remitter, did not significantly differ with regard to type or frequency of depression treatment during follow-up (Supplementary Table S5).

### 3.2 Impact of remission on symptoms of depression and anxiety

**Figure 1:**
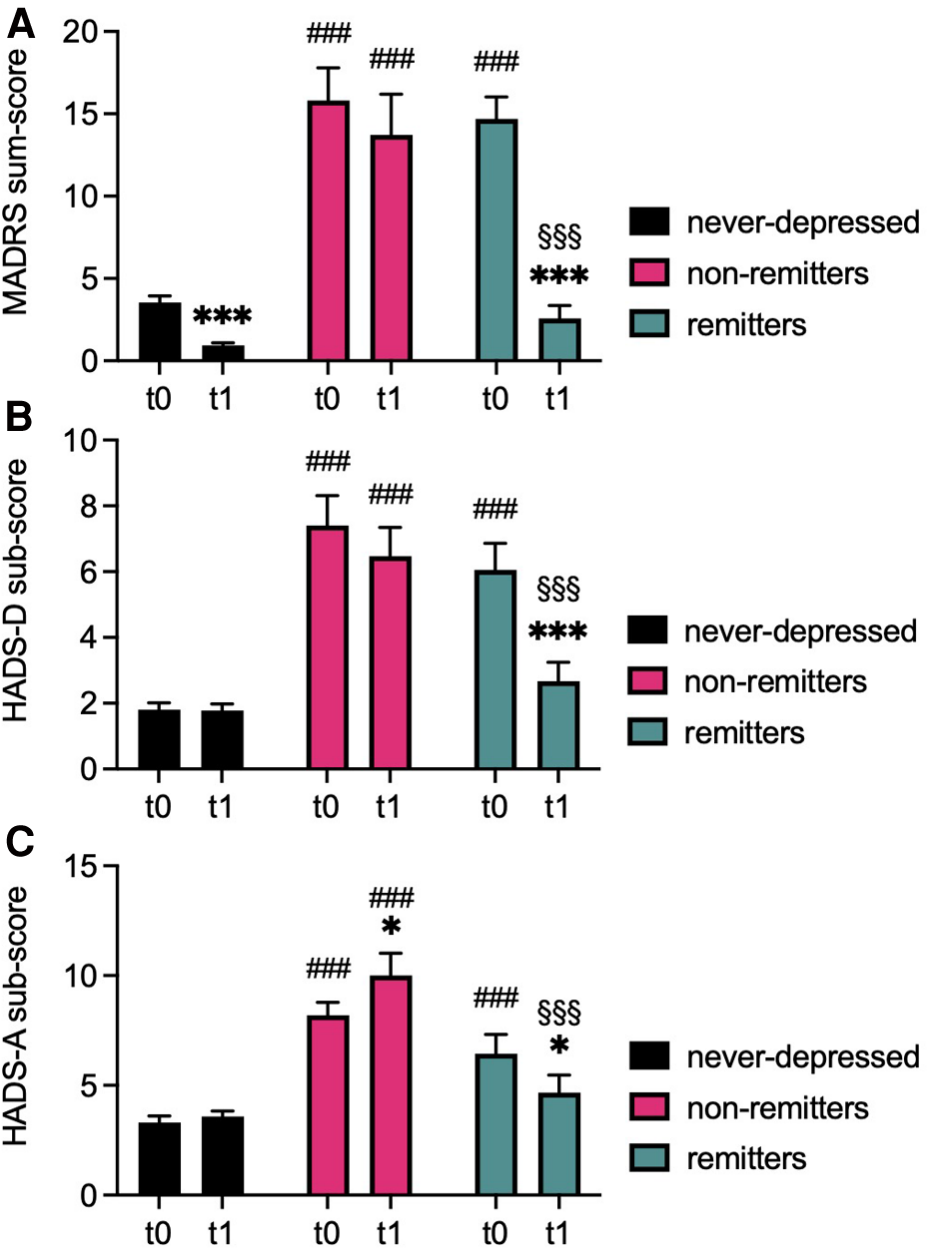
Severity of depression and anxiety at baseline and follow-up. Bargraphs depict mean ± standard error of means (SEM) of clinician-rated (MADRS, **A**) and self-rated (HADS-D, **B**) depression scores and of self-rated symptoms of anxiety (HADS-A, **C**) of never-depressed, non-remitted, and remitted ACHD at baseline (t0) and follow-up (t1). ***P < 0.001, *P < 0.05 vs. corresponding t0 of the same group, ###*P* < 0.001 vs. corresponding timepoint never-depressed group, §§§*P* < 0.001 vs. corresponding timepoint chronic depression group. *P* < 0.05 was considered statistically significant.

Data regarding measures of depression and anxiety are summarized in Table 1, Supplementary Tables S6, S7, and Figure 1. Repeated measures ANOVA indicated a significant interaction effect on clinician-rated depression score [MADRS*: F*(2, 111) = 30.442, *P* < 0.001] as well as on the self-rated depression sub-scale of the HADS [HADS-D: *F*(2, 111) = 10.336, *P* < 0.001]. Similarly, significant interaction effects on the anxiety sub-scale of the HADS [HADS-A: *F*(2, 111) = 6.097, *P* = 0.003] were detected.

**Table 1:** Severity of depression and anxiety symptoms at baseline and follow-up.

	Never-depressed			Non-remitters			Remitters		
	t0	t1	*P*-value	t0	t1	*P*-value	t0	t1	*P*-value
MADRS sum-score	3.5 ± 3.6	0.9 ± 1.4	<0.001	15.8 ± 7.7	13.7 ± 9.5	0.098	14.7 ± 5.7	2.6 ± 3.3	<0.001
HADS-D sub-score	1.8 ± 1.9	1.8 ± 1.8	0.938	7.4 ± 3.5	6.5 ± 3.4	0.207	6.1 ± 3.4	2.7 ± 2.4	<0.001
HADS-A sub-score	3.3 ± 2.7	3.6 ± 2.3	0.415	8.2 ± 2.2	10.0 ± 3.9	0.021	6.4 ± 3.7	4.7 ± 3.4	0.013

Mean ± standard deviation of clinician-rated (MARDS) and self-reported (HADS-D) depression scores and self-rated anxiety symptoms (HADS-A) are depicted. Baseline (t0) and follow-up (t1) data are shown. Bonferroni-corrected *P*-values relate to changes between t0 and t1 within the respective groups. *P* < 0.05 was considered statistically significant. HADS, Hospital Anxiety and Depression Scale; MADRS, Montgomery-Asperg Depression Rating Scale.

Analysis showed significant main effects for group for MADRS [*F*(2, 111) = 92.151, *P* < 0.001], for the depression subscale of the HADS [HADS-D: *F*(2, 111) = 57.747, *P* < 0.001], and for the HADS anxiety subscale [HADS-A: *F*(2, 111) = 39.163, *P* < 0.001]. Similarly, significant main effects for time were detected for MADRS [*F*(1, 111) = 91.107, *P* < 0.001] and for HADS-D [*F*(1, 111) = 17.296, *P* < 0.001], but not for HADS-A [*F*(1, 111) = 0.072, *P* = 0.789].

Pairwise comparisons at t0 showed significantly higher clinician-rated and self-rated depression scores in both depression groups compared to never-depressed patients (all *P*-values <0.001). Similarly, anxiety scores were significantly higher in both depression groups compared to never-depressed (all *P*-values <0.001). Additionally, both depression groups did not significantly differ with regard to depression or anxiety scores at t0 (all *P*-values ≥0.229).

At t1, non-remitters continued to report significantly increased depression and anxiety scores compared to never-depressed patients as well as compared to remitters (all *P*-values <0.001). No significant differences were evident regarding depression and anxiety scores when comparing remitters to never-depressed patients at t1 (all *P*-values ≥0.320).

Clinician-rated depression scores improved significantly in the remitter group (MADRS: *P* < 0.001), but also in never-depressed patients (MADRS: *P* < 0.001) between t0 and t1. Self-rated depression symptoms improved significantly only in the remitter group (HADS-D: *P* < 0.001). No significant change in clinician-rated or self-reported depression scores was observed in the non-remitter group (MADRS: *P* = 0.098, HADS-D: *P* = 0.207). Anxiety scores did not significantly change in never-depressed patients (*P* = 0.415), increased in the non-remitter group (*P* = 0.021), and significantly decrease in the remitter group (*P* = 0.013).

### 3.3 Impact of remission on health-related quality of life

**Figure 2:**
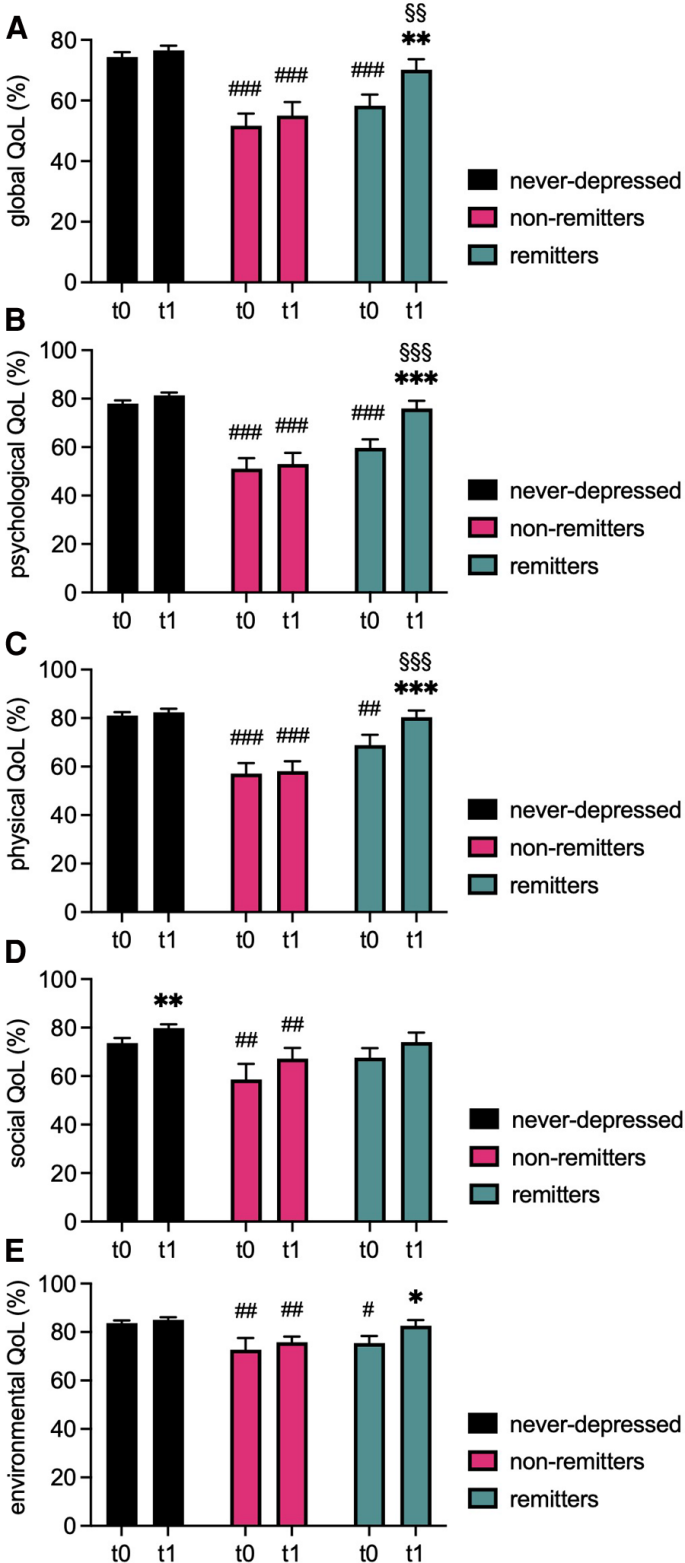
Comparison of QoL domains at baseline and follow-up. Bargraphs depict mean ± standard error of means (SEM) of global quality of life (QoL, **A**) and of the psychological (**B**), physical (**C**), social (**D**), and environmental (**E**) domain scores of the WHOQOL-BREF questionnaire of never-depressed, non-remitted, and remitted ACHD at baseline (t0) and follow-up (t1). ***P < 0.001, **P < 0.01, *P < 0.05 vs. corresponding t0 of the same group, ###*P* < 0.001, ##*P* < 0.01, #*P* < 0.05 vs. corresponding timepoint never-depressed group, §§§*P* < 0.001, §§*P* < 0.01 vs. corresponding timepoint chronic depression group. *P* < 0.05 was considered statistically significant.

Results regarding QoL (WHOQOL-BREF) are depicted in Table 2, Supplementary Tables S8, S9, and in Figure 2. Repeated measures ANOVA failed to show a significant interaction effect for global WHOQOL score [*F*(2, 111) = 2.881, *P* = 0.060] or for its social domain [*F*(2, 111) = 0.121, *P* = 0.886] and environmental domain [*F*(2, 110) = 1.830, *P* = 0.165]. However, significant interaction effects were detected for the WHOQOL physical [*F*(2, 111) = 3.997, *P* = 0.021] and psychological [*F*(2, 111) = 7.582, *P* < 0.001] domains.

**Table 2:** Quality of life at baseline and follow-up.

	Never-depressed			Non-remitters			Remitters		
	t0	t1	*P*-value	t0	t1	*P*-value	t0	t1	*P*-value
QoL global sumscore (%)	74.4 ± 14.1	76.5 ± 13.7	0.211	51.7 ± 15.6	55.0 ± 17.6	0.405	58.3 ± 15.5	70.1 ± 14.9	0.002
QoL physical subscore (%)	81.0 ± 12.6	82.4 ± 13.3	0.384	57.1 ± 16.6	58.1 ± 15.8	0.794	68.8 ± 18.2	80.4 ± 11.6	<0.001
OoL psychological subscore (%)	78.0 ± 11.7	81.4 ± 10.3	0.022	51.1 ± 16.8	53.1 ± 17.6	0.567	59.7 ± 14.8	75.9 ± 13.6	<0.001
QoL social subscore (%)	73.7 ± 18.2	79.8 ± 14.3	0.003	58.6 ± 24.8	67.2 ± 17.1	0.066	67.6 ± 16.9	74.1 ± 16.6	0.129
QoL environmental subscore (%)	83.7 ± 9.7	85.0 ± 10.3	0.317	72.7 ± 18.9	75.8 ± 8.9	0.308	75.4 ± 12.5	82.6 ± 10.0	0.011

The short form of the World-Health Organization Quality of Life (QoL) questionnaire (WHOQoL-BREF) was used. Data are depicted as mean ± standard deviation. Baseline (t0) and follow-up (t1) data are depicted. Bonferroni-corrected *P*-values relate to changes between t0 and t1 within the respective groups. *P* < 0.05 was considered statistically significant.

Significant main effects for group were found for global QoL [*F*(2, 111) = 23.673, *P* < 0.001], and for all individual domains (physical: *F*(2, 111) = 26.888, *P* < 0.001; psychological: *F*(2, 111) = 46.499, *P* < 0.001; social: *F*(2, 111) = 6.170, *P* = 0.003; environmental: *F*(2, 110) = 8.810, *P* < 0.001). Additionally, a significant main effect for time was found for global QoL [*F*(1, 111) = 9.323, *P* = 0.003] and for all individual domains (physical: *F*(1, 111) = 20.054, *P* < 0.001; physiological: *F*(1, 111) = 7.184, *P* = 0.008; social: *F*(1, 111) = 10.342, *P* = 0.002; environmental: *F*(1, 110) = 7.194, *P* = 0.008).

Pairwise comparisons at t0 indicated that non-remitters had significantly lower scores for global QoL as well as for all individual domains compared to never-depressed patients (global: (*P* < 0.001); physical: (*P* < 0.001); psychological: (*P* < 0.001); social: (*P* = 0.017); environmental: (*P* = 0.004). Similarly, remitters displayed significantly lower values for global QoL as well as for most individual domains compared to never-depressed patients at t0 (global: *P *< 0.001; physical: *P *= 0.004; psychological: *P *< 0.001; environmental: *P *= 0.025). Only the social domain did not significantly differ between both groups at t0 (*P* = 0.668). No significant group differences were observed between both depression groups regarding measures of QoL at t0 (all *P*-values ≥0.058).

At t1, reported QoL remained poor in the non-remitter group compared to never-depressed patients (global: *P *< 0.001; physical: *P *< 0.001; psychological: *P *< 0.001; social: *P *= 0.011; environmental: *P *= 0.005). Contrarily, no significant differences were observed regarding global QoL (*P* = 0.276), or any of the individual domains (all *P*-values ≥0.253) when comparing remitters to never-depressed patients. Accordingly, at t1, remitters scored higher in global QoL (*P* = 0.010), physical QoL (*P* < 0.001), and physiological QoL (*P* < 0.001) compared to non-remitted patients.

Global QoL improved significantly in the remitter group between t0 and t1 (*P* = 0.002), and similarly significant improvement on all but one domain was observed (physical: *P *< 0.001; psychological: *P *< 0.001; environmental: *P* = 0.011). Contrarily, the non-remitter group showed no significant time-dependent change in global QoL (*P* = 0.405) or in any of the respective domains of the WHOQOL (all *P*-values ≥0.066). Never-depressed patients reported significantly higher values in the psychological (*P* = 0.022) and the social (*P* = 0.003) domain, while global QoL (*P* = 0.211), physical (*P* = 0.384), and environmental (*P* = 0.317) domains did not significantly change.

### 3.4 Impact of remission on cardiorespiratory function

**Figure 3:**
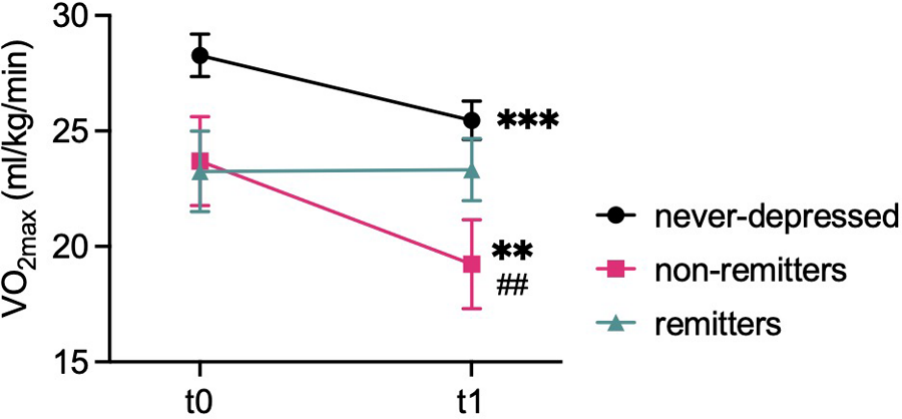
Development of exercise capacity. Graph depicts mean ± standard error of means (SEM) of exercise capacity (VO_2max_) at baseline (t0) and follow-up (t1) of never-depressed, non-remitted, and remitted ACHD patients at either timepoint. ***P < 0.001, **P < 0.01 vs. corresponding t0 of the same group, #*P* < 0.05 vs. corresponding timepoint of the never-depressed group. *P* < 0.05 was considered statistically significant.

Parameters of cardiorespiratory function are summarized in Table 3 and Supplementary Tables S10, S11. Further, data regarding VO_2max_ are depicted in Figure 3.

**Table 3:** Development of exercise capacity, health behavior, and cardiovascular risk factors at baseline and follow-up.

	Never-depressed			Non-remitters			Remitters		
	t0	t1	*P*-value	t0	t1	*P*-value	t0	t1	*P*-value
BMI (kg/$m^{2}$)	24.1 ± 3.7	24.8 ± 4.1	0.002	27.7 ± 4.8	29.2 ± 5.3	0.004	24.1 ± 2.6	24.7 ± 3.0	0.220
Drinks/week	2.2 ± 3.5	1.8 ± 2.5	0.351	0.6 ± 1.1	1.1 ± 1.5	0.660	0.7 ± 1.2	3.0 ± 9.8	0.020
Sport	3.64 ± 1.56	3.71 ± 1.41	0.715	2.92 ± 1.38	2.85 ± 1.34	0.861	3.03 ± 1.50	3.65 ± 1.62	0.094
VO_2max_ (ml/kg/min)	28.3 ± 7.6	25.5 ± 7.0	<0.001	23.7 ± 6.4	19.9 ± 6.1	0.006	23.2 ± 6.5	23.3 ± 5.0	0.958
WR ind (W/kg)	2.16 ± 0.60	2.11 ± 0.56	0.219	1.74 ± 0.56	1.56 ± 0.49	0.044	1.86 ± 0.64	1.93 ± 0.50	0.433
LVEF (%)	56.7 ± 8.7	57.7 ± 7.9	0.250	59.0 ± 8.3	56.9 ± 8.1	0.242	59.1 ± 11.6	56.8 ± 8.7	0.200
LVEDD (mm)	54.5 ± 7.7	54.5 ± 6.1	0.899	55.1 ± 5.2	54.2 ± 5.6	0.493	50.9 ± 4.8	52.0 ± 3.6	0.372
NT-proBNP (ng/L)	207 ± 296	221 ± 266	1.000	202 ± 261	263 ± 210	1.000	161 ± 184	208 ± 237	1.000
CRP (mg/L)	3.5 ± 6.6	2.4 ± 3.3	0.144	6.1 ± 9.1	3.2 ± 2.2	0.087	2.5 ± 3.5	1.3 ± 0.9	0.403
NYHA class	1.23 ± 0.54	1.29 ± 0.56	0.319	1.40 ± 0.63	1.60 ± 0.74	0.092	1.33 ± 0.59	1.33 ± 0.59	1.000

Continuous variables are depicted as mean ± standard deviation and categorical variables are shown as percentages as indicated. Baseline (t0) and follow-up (t1) data are depicted. Bonferroni-corrected *P*-values relate to changes between t0 and t1 within the respective groups. *P* < 0.05 was considered statistically significant. BMI, body mass index; CRP, C-reactive protein; LVEDD, left ventricular end-diastolic parameter; LVEF, left ventricular ejection fraction; NT-proBNP, N-terminal prohormone of brain natriuretic peptide; NYHA, New-York Heart Association Classification; VO_2max_, maximum oxygen uptake; WR ind maximum work rate indexed to bodyweight.

Repeated measures ANOVA failed to detect a significant interaction effect for VO_2max_ [*F*(2, 92) = 2.580, *P* = 0.081] or for maximum work rate indexed to bodyweight [WR: *F*(2, 96) = 2.037, *P* = 0.136].

Significant main effects for group regarding VO_2max_ [*F*(2, 92) = 4.414, *P* = 0.015] as well as WR [*F*(2, 96) = 5.056, *P* = 0.008] were detected. Further, a significant main effect for time was found for VO_2max_ [*F*(1, 92) = 10.713, *P* = 0.001], but not for WR [*F*(1, 96) = 1.581, *P* = 0.212].

Pairwise comparisons indicated no statistically significant group differences regarding VO_2max_ (all *P*-values ≥0.066) or WR (all *P*-values ≥0.062) at t0. However, non-remitters displayed significantly lower VO_2max_ (*P* = 0.015) and WR (*P* = 0.002) values compared to never-depressed patients at t1. No significant differences in VO_2max_ or WR were observed between the remitter group and never-depressed group (VO_2max_: *P* = 0.829, WR: *P* = 0.735) and between remitters and non-remitters (VO_2max_: *P* = 0.391, WR: *P* = 0.213) at t1.

A significant decrease in VO_2max_ over time was observed in never-depressed patients (*P* < 0.001) and in non-remitters (*P* = 0.006). Additionally, WR decreased significantly in the non-remitter group (*P* = 0.044). Contrarily, VO_2max_ and WR were preserved in the remitter group (VO_2max_: *P* = 0.958; WR: *P* = 0.433) and no significant change in WR was observed in never-depressed patients (*P* = 0.219).

### 3.5 Impact of remission on cardiovascular risk markers and health behaviours

Results concerning cardiovascular risk parameters and health behaviours are summarized in Table 3 as well as in Supplementary Tables S10, S11.

With regard to cardiac left ventricular (LV) function (ejection fraction, LVEF), and LV dimensions (LV end-diastolic parameter, LVEDD), repeated measures ANOVA showed no significant interaction effect (LVEF: *F*(2, 109) = 2.191, *P* = 0.117; LVEDD: *F*(2, 110) = 0.623, *P* = 0.538) and no significant main effects for group (LVEF: *F*(2, 109) = 0.102, *P* = 0.903; LVEDD: *F*(2, 110) = 2.103, *P* = 0.127) or for time (LVEF: *F*(1, 109) = 1.688, *P* = 0.197; LVEDD: *F*(1, 110) = 0.017, *P* = 0.896) were detected.

Further, no significant interaction effect, main effect for group, or for time was found for functional heart failure status, indicated by NYHA class (interaction effect: *F*(2, 107) = 0.874, *P* = 0.420; group effect: *F*(2, 107) = 1.333, *P* = 0.268; time effect: *F*(1, 107) = 2.264, *P* = 0.135), or for levels of the prognostic biomarker NT-proBNP (interaction effect: *F*(2, 108) = 0.613, *P* = 0.544; group effect: *F*(2, 108) = 0.153, *P* = 0.859; time effect: *F*(1, 108) = 3.629, *P* = 0.059).

Additionally, repeated measures ANOVA showed no significant interaction effect [*F*(2, 109) = 0.495, *P* = 0.611] or main effect for group [*F*(2, 109) = 2.038, *P* = 0.135] for CRP levels. While a significant main effect for time [*F*(1, 109) = 4.847, *P* = 0.030] was found, pairwise comparisons failed to detect significant changes in CRP in any of the study groups between t0 and t1 (all *P*-values ≥0.087).

Contrarily, while repeated measures ANOVA showed no significant interaction effect for BMI [*F*(2, 111) = 1.149, *P* = 0.321], a significant main effect for group [*F*(2, 111) = 7.564, *P* = 0.002] and for time [*F*(1, 111) = 14.420, *P* < 0.001] was detected.

Respective pairwise comparisons showed that compared to never-depressed patients, non-remitters displayed significantly higher BMI values at t0 (*P* = 0.002) as well as at t1 (*P* < 0.001). Further, non-remitters presented with significantly higher BMI values compared to remitters at both timepoints (t0: *P* = 0.016; t1: *P* = 0.006). Conversely, no significant differences were observed between never-depressed patients and remitters (t0: *P* = 1.00; t1: *P* = 1.00). Further, BMI values in never-depressed patients (*P* = 0.002) as well as in non-remitters (*P* = 0.004), increased over time, while no significant change in BMI was observed in the remitter group (*P* = 0.220).

With regard to health behaviour, repeated measures ANOVA failed to detect a significant interaction effect (*F*(2, 102) = 1.084, *P* = 0.342, main effect for group [*F*(2, 102) = 2.424, *P* = 0.094], or main effect for time [*F*(1, 102) = 1.095, *P* = 0.298] with regard to subjective, self-rated physical exercise scores. However, pairwise comparisons indicated a trend towards increased physical activity in the remitter group over time (*P* = 0.094), while no such effect was observed in the other study groups (never- depressed: *P* = 0.715; non-remitters: *P* = 0.861).

Further, a significant interaction effect was found for alcohol consumption [*F*(2, 111) = 3.255, *P* = 0.042], but no significant main effect for group [*F*(2, 111) = 0.899, *P* = 0.410] or time [*F*(1, 111) = 2.366, *P* = 0.127] was detected. Pairwise comparisons showed a significant increase in alcohol consumption over time in the remitter group (*P* = 0.020), but not in the other study groups (never-depressed: *P* = 0.361; non-remitters: *P* = 0.600).

Finally, smoking status changed over time. Chi-square test indicated significant differences between groups at t0 [*χ*^2^(2) = 8.8, *P* = 0.033], with remitters reporting the highest smoking frequency (t0: 50%), compared to non-remitters (t0: 20%), and never-depressed patients (t0: 21%). At t1, no significant group differences were detected [*χ*^2^(2) = 0.99, *P* = 0.600]. Of note, a marked decline in smoking frequency was observed in remitters (t1:17%), while the rate of smokers remained stable in never-depressed patients (t1: 15%) and increased slightly in non-remitters (t1: 27%).

## 4 Discussion

To the best of our knowledge, this is the first prospective observational study that evaluated the impact of time dependent changes in depression status on anxiety symptoms, QoL, and exercise capacity in ACHD patients. Our data indicate that remission from depression was associated with improved anxiety levels and better QoL. Furthermore, exercise capacity was preserved in remitted patients and beneficial effects on BMI were observed. Contrarily, non-remission was associated with persistently increased anxiety scores and poor QoL, decreased exercise capacity and higher BMI values.

The observed improvement in mental health in the remitter group was associated with a significant increase in overall QoL as well as improvement in the respective domains of the WHOQOL-BREF. Our results are in line with data from psychiatric outpatients suffering from major depressive disorder that indicate depressive symptom severity, functional impairment and QoL to be highly intercorrelated parameters (39). In the context of cardiovascular disease, depressive symptoms have been shown to mediate the relationship between health-related QoL and cardiac event-free survival (40) and depression has been established as the main driver of QoL in various cardiovascular patient populations (23, 41, 42). Furthermore, QoL has been an established marker for morbidity and mortality in patients with chronic heart failure (43, 44) and the importance of health-related QoL in patients with cardiovascular disease is highlighted by studies reporting that patients with heart failure citing QoL to be similarly or more important to them than longevity (9, 10).

Both depression and anxiety have been shown to be associated with exercise capacity in cardiovascular disease patients (22, 45). A dedicated meta-analysis that included 59 studies with over 25,000 heart disease patients concluded that increased depressive symptoms may be associated with reduced exercise capacity and vice versa (46). Additionally, the authors highlighted that the impact of depression improvement on exercise capacity should be further explored (46). This is of importance as decreased exercise capacity has been demonstrated to correlate with hospitalization and mortality in ACHD patients (16).

No statistically significant group differences in VO_2max_ were observed between depressed and non-depressed patients at baseline, although values tended to be decreased in depressed patients compared to non-depressed patients. This observation is in line with the previously reported association of exercise capacity and depressive symptomology in cardiovascular patients (22, 45). Importantly, in the present study, exercise capacity at follow-up was persevered only in ACHD patients that remitted from depression. Contrarily, exercise capacity declined in never-depressed- as well as in non-remitted patients. This observed “natural decline” in cardiorespiratory function is in line with prior observations, reporting a large variability in deteriorating maximum exercise capacity independent from cardiac function (47–49). Finally, our data are in line with previous studies, indicating decreased exercise capacity to be associated with low QoL scores in various cardiovascular patient populations, including patients with ACHD (17, 50).

Several factors have been discussed to adversely impact exercise capacity in patients with cardiovascular disease. In the present sample, parameters indicative of current heart disease severity, including NYHA class, cardiac function, and cardiac biomarker profiles did not significantly differ between groups based on depression status, neither at baseline nor at follow-up. Further, no significant change in these parameters was observed over time. Our data are congruent with previous work reporting that severity of the underlying heart condition and present symptoms of heart disease are not predictive of depression in cardiovascular patient populations including ACHD patients (24). However, an increased long-term risk for depression has been described for patients with complex lesions (51). This discrepancy might be explained by the included high proportion of patients with complex cardiac lesions.

Similarly, meta-analytical data suggests that changes in exercise capacity in response to exercise training over time appear independent of changes in left ventricular systolic and diastolic function in heart failure patients (52). Additionally, exercise training in patients with a systemic right ventricle was found to improve exercise capacity without impacting levels of NTpro-BNP (53).

In line with the described beneficial impact of physical activity on exercise capacity in ACHD patients, in the present study, preserved exercise capacity in the remitter group was associated with increased self-reported physical activity by trend. In this regard, the adverse impact of major depressive disorder on physical activity has been well established in the general population as well as in cardiovascular disease patients (30, 54). Additionally, studies have shown that daily activity, which has been shown to be diminished in the context of depression (55), correlated with exercise capacity in ACHD patients (56).

Next to physical exercise, our data confirm a marked improvement in other lifestyle factors in remitted patients that was not observed in non-remitters. The observed percentage of smokers in the present sample was comparable to previous studies by others (57, 58). In the present study, the rate of smokers declined, and the BMI remained stable in the remitter group, while a significant increase in BMI values was observed non-remitters at the five years follow-up. Both, smoking and heightened BMI, have been reported to be associated with lower levels of regular physical exercise, and conversely exercise has been described as an intervention for weight management and smoking cessation (59, 60).

Taken together our data point towards an improvement in mental well-being in ACHD patients, who remitted from depression compared to those, who did not. Similar to data derived from other cardiac patient populations as well as from the general population, this appears to be associated with improvements in lifestyle behaviours, including physical exercise and smoking, which constitute well-established cardiac risk factors and have been previously linked to adverse developments in exercise capacity (56). Finally, both remission from depression itself and the associated improvement in mental well-being, as well as preserved exercise capacity, might impact QoL, particularly with regard to the physical and psychological domains (23, 39, 41, 42).

### 4.1 Clinical implications

Our results highlight the importance of identifying patients with ACHD, who are comorbid with mental disorders, particularly major depression. According to recent guidelines of the European Society of Cardiology (ESC), a screening for depression in patients with cardiovascular disease is recommended (61–63). Several reliable instruments have been developed so far. The standard for research is the Structured Clinical Interview for DSM-5 (64). However, this interview is time consuming and needs specific training. Screening instruments such as the Beck depression Inventory-2 (BDI-2) and the HADS have also been recommended (33, 65). For the BDI-2 and HADS, lower thresholds have been recommended when used in patients with ACHD (34).

While data regarding long-term remission from depression in cardiovascular patients are lacking, data from psychiatric samples commonly report remission rates of over 80% (66, 67). Compared to these data, the remission rate in our sample of depressed ACHD patients was lower, with only 55% reaching remission at the 5-year follow-up. This finding is clinically important and may point to regular monitoring of depressed ACHD patients in a multimodal treatment plan.

Considering the beneficial effects of remission from depression not only on mental health parameters but also on overall QoL and exercise capacity, which both have been demonstrated to be of prognostic relevance in cardiovascular patients as well as in patients ACHD (15, 16, 68–70), our data highlight the importance of optimizing not only diagnosis but also treatment of depression in these patients. In this regard, exercise training that denotes an integral part of depression treatment in psychiatric patients as well as in patients with other heart diseases, might be considered as an important intervention in ACHD, provided that the individual cardiac risk constellation is taken into account (49, 70–72).

## 5 Limitations

There are some limitations that should be taken into account when interpreting the presented data. Although the complete sample included over *N* = 100 patients, sample sizes in the respective depression groups are modest and results should be confirmed in a greater sample.

Further, the observational nature of our study does preclude any causal inferences and additional factors not assessed in the present study might critically contribute to observed changes and differences, particularly with regard to health-related QoL and exercise capacity. Additionally, next to the reported sociodemographic data, additional factors have been discussed to influence particularly exercise capacity but also QoL in ACHD patients. In this regard, socioeconomic status has been described to be associated with exercise capacity in these patients (73) Further, the assessment of physical activity was carried out with a self-rating score. Further studies with larger patient groups might consider the use of wearables for a more objective measurement of physical activity and sport.

## 6 Conclusion

Our results demonstrate that remission from depression is associated with significant improvement concerning mental health, QoL, lifestyle factors and with a maintenance of exercise capacity. Together with the finding that non-remitters continued to report low mental well-being and QoL scores as well as a further decline in exercise capacity, which represents a well-established prognostic marker in ACHD (16), our results highlight the importance of adequate diagnosis and treatment, including follow-up visits, of mental health conditions, particularly of depression, in this patient population. ESC guidelines recommend that ACHD centres should provide a minimum of one psychologist. Further, screening for symptoms of depression and anxiety is recommended using established questionnaires. We have observed in other studies, that depression is a significant contributor of non-adherence and cardiovascular mortality (74). Therefore, optimizing the identification and treatment of major mental disorders, particularly major depression, is recommended as part of a multimodal treatment plan in ACHD.

## Data Availability

The raw data supporting the conclusions of this article will be made available by the authors, without undue reservation.
