# Supplementary material for: Remission from depression is associated with improved quality of life and preserved exercise capacity in adults with congenital heart disease
Source: Front Cardiovasc Med. 2024 Jul 3;11:1418342. doi: 10.3389/fcvm.2024.1418342 (PMC11251921; doi:10.3389/fcvm.2024.1418342)
Supplement: Supplementary file 1 [file Datasheet1.pdf]

Supplementary Material

1 Supplementary Figures

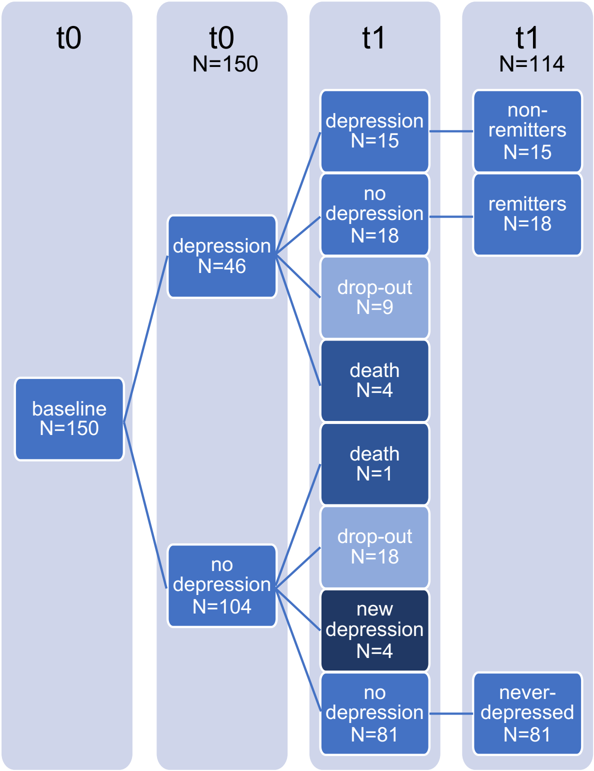

Supplementary Figure 1. Group affiliation of patients. Group affiliation of ACHD patients at baseline (t0) and at follow-up (t1). N-numbers of ACHD patients that were diagnosed with major depression at t0 and t1, as well as N-numbers of patients that dropped out in either group are depicted.

2 Supplementary Tables

Supplementary Table 1: Baseline characteristics of the complete study sample.

	Complete sample at t0 (N=114)
Age (years)	34.3±11.5
Male/female sex [N (%)]	63 (54%) / 51 (47%)
Weight (kg)	75.7±15.3
BMI (kg/m^2)	25.0±6.0
NYHA (I/II/III) [N (%)]	90 (79%) / 18 (16%) / 6 (5%)
Bethesda (I/II/III) [N (%)]	64 (56%) / 30 (26%) / 20 (18%)
LVEF (%)	57.4±9.2
LVEDD (mm)	54.0±7.1
NT-proBNP (ng/l)	199.3±275.2
CRP (mg/l)	1.6±1.3
VO_{2max} (ml/kg/min)	26.7±7.8
WR ind (W/kg, N=100)	2.05±0.62
RR (mmHg)	111.1±15.0
RRmax (mmHg)	168.1±30.3
MADRS sumscore	6.9±7.0
HADS-D subscore	3.2±3.3
HADS-A subscore	4.5±3.3
QoL global sumscore (%)	68.9±16.9

Continuous variables are depicted as mean ± standard deviation and categorical variables are shown as numbers (N) and/or percentages as indicated. BMI, body mass index; CRP, C-reactive protein; HADS, Hospital Anxiety and Depression Scale; HADS-A, HADS anxiety subscale; HADS-D, HADS depression subscale; LVEDD, left ventricular end-diastolic diameter; LVEF, left ventricular ejection fraction; MADRS, Montgomery-Asperg Depression Rating Scale; NT-proBNP, N-terminal prohormone of brain natriuretic peptide; NYHA class, New-York Heart Association Classification; RR, resting blood pressure; RRmax, maximum blood pressure; VO_{2max}, maximum oxygen uptake; WR ind maximum work rate indexed to body-surface area

Supplementary Table 2: Overview regarding complexity and type of the underlying congenital heart defect as well as pharmacologic treatment of the cardiovascular condition of the complete sample at baseline

	Complete sample at t0 (N=114)
Bethesda class (I/II/III)	64 (56%) / 30 (26%) / 20 (18%)
Coarctation	16 (14%)
Tetralogy of Fallot	16 (14%)
Bicuspid aortic valve	15 (13%)
D-transposition: atrial switch	13 (12%)
Simple shunts	10 (9%)
Fontan type circulation	9 (8%)
Marfan syndrome	8 (7%)
Congenital pulmonary stenosis	7 (6%)
Atrioventricular septal defect	5 (5%)
Subaortic stenosis	3 (3%)
Eisenmenger syndrome	2 (2%)
Anomalous pulmonary venous connection	1 (1%)
Common arterial trunc	1 (1%)
Congenital corrected transposition	1 (1%)
D-transposistion: arterial switch	1 (1%)
Double chambered right ventricle	1 (1%)
Ebstein anomaly	1 (1%)
Mitral valve disease	1 (1%)
Subaortic stenosis	1 (1%)
Supravalvular aortic stenosis	1 (1%)
ACE/AT-inhibitor	50 (44%)
Beta-blockers	31 (27%)
Ca-antagonist	4 (4%)
Diuretics	11 (10%)
Mineralocorticoid receptor antagonist	10 (9%)
Vitamin K antagonist	26 (23%)

Data are depicted as number and percentages [N (%)] and related to the complete sample of N = 114 ACHD patients at baseline (t0). ACE, angiotensin-converting enzyme; AT, angiotensin type receptor.

Supplementary Table 3: Group comparison concerning the complexity and type of the underlying congenital heart condition at baseline.

	nerver-depressed	non-remitters	remitters	Statistical analysis
Bethesda class	I: 47 (58%)<br>II: 20 (25%)<br>III: 14 (17%)	I: 7 (48%)<br>II: 6 (40%)<br>III: 2 (13%)	I: 10 (56%)<br>II: 4 (22%)<br>III: 4 22%)	$\chi^2(4) = 1.935$, $P = 0.748$
Coarctation	10 (13%)	4 (27%)	2 (11%)	$\chi^2(2) = 2.303$, $P = 0.316$
Tetralogy of Fallot	12 (15%)	1 (7%)	3 (17%)	$\chi^2(2) = 0.819$, $P = 0.664$
Bicuspid aortic valve	11 (14%)	3 (20%)	1 (6%)	$\chi^2(2) = 1.538$, $P = 0.464$
D-transposition: atrial switch	6 (8%)	2 (13%)	5 (28%)	$\chi^2(2) = 6.112$, $P = 0.047$
Simple shunts	7 (9%)	1 (7%)	2 (11%)	$\chi^2(2) = 0.208$, $P = 0.901$
Fontan type circulation	6 (8%)	2 (13%)	1 (6%)	$\chi^2(2) = 0.772$, $P = 0.680$
Marfan syndrome	7 (9%)	0 (0%)	1 (6%)	$\chi^2(2) = 1.519$, $P = 0.468$
Congenital pulmonary stenosis	4 (5%)	1 (7%)	2 (11%)	$\chi^2(2) = 0.982$, $P = 0.612$
Atrioventricular septal defect	4 (5%)	1 (7%)	0 (0%)	$\chi^2(2) = 1.071$, $P = 0.585$
Subaortic stenosis	2 (3%)	0 (0%)	0 (0%)	$\chi^2(2) = 0.829$, $P = 0.661$
Eisenmenger syndrome	2 (3%)	0 (0%)	0 (0%)	$\chi^2(2) = 0.829$, $P = 0.661$
Anomalous pulmonary venous connection	1 (1%)	0 (0%)	0 (0%)	$\chi^2(2) = 0.411$, $P = 0.814$
Common arterial trunc	1 (1%)	0 (0%)	0 (0%)	$\chi^2(2) = 0.411$, $P = 0.814$
Congenital corrected transposition	1 (1%)	0 (0%)	0 (0%)	$\chi^2(2) = 0.411$, $P = 0.814$
D-transposistion: arterial switch	1 (1%)	0 (0%)	0 (0%)	$\chi^2(2) = 0.411$, $P = 0.814$
Double chambered right ventricle	1 (1%)	0 (0%)	0 (0%)	$\chi^2(2) = .411$, $P = 0.814$
Ebstein anomaly	1 (1%)	0 (0%)	0 (0%)	$\chi^2(2) = 0.411$, $P = 0.814$
Mitral valve disease	1 (1%)	0 (0%)	0 (0%)	$\chi^2(2) = 0.411$, $P = 0.814$
Subaortic stenosis	2 (3%)	0 (0%)	1 (6%)	$\chi^2(2) = 1.014$, $P = 0.602$
Supravalvular aortic stenosis	1 (1%)	0 (0%)	0 (0%)	$\chi^2(2) = 0.411$, $P = 0.814$

Data are depicted as number and percentages [N (%)] and related to the respective depression groups at baseline (t0). Chi-square test was applied and two-tailed *P*-values are depicted. $P < 0.05$ was considered statistically significant.

Supplementary Table 4: Group comparison regarding pharmacologic treatment of the cardiovascular condition of ACHD patients at baseline.

	nerver-depressed	non-remitters	remitters	Statistical analysis
ACE/AT-inhibitor	39 (48%)	6 (40%)	5 (30%)	$\chi^2(2) = 2.586$, $P = 0.274$
Beta-blockers	22 (28%)	6 (40%)	3 (17%)	$\chi^2(2) = 2.238$, $P = 0.327$
Ca-antagonist	3 (4%)	1 (7%)	0 (0%)	$\chi^2(2) = 1.105$, $P = 0.575$
Diuretics	6 (7%)	3 (20%)	2 (11%)	$\chi^2(2) = 2.354$, $P = 0.308$
Mineralocorticoid receptor antagonist	7 (9%)	1 (7%)	2 (11%)	$\chi^2(2) = 0.208$, $P = 0.901$
Vitamin K antagonist	19 (24%)	4 (27%)	3 (17%)	$\chi^2(2) = 0.532$, $P = 0.767$

Data are depicted as number and percentages [N (%)] and related to the respective depression groups at baseline (t0). Chi-square test was applied and two-tailed *P*-values are depicted. $P < 0.05$ was considered statistically significant. ACE, angiotensin-converting enzyme; AT, angiotensin type receptor.

Supplementary Table 5: Depression treatment over the follow-up period in ACHD with a depression diagnosis at baseline.

	non-remitters	remitters	Statistical analysis
No therapy	7 (47%)	10 (56%)	$\chi^2(1) = 0.259$, $P = 0.611$
Psychotherapy only	4 (27%)	4 (22%)	$\chi^2(1) = 0.088$, $P = 0.767$
Psychopharmacotherapy only	2 (13%)	1 (6%)	$\chi^2(1) = 0.599$, $P = 0.439$
Psychotherapy and Psychopharmacotherapy	2 (13%)	3 (17%)	$\chi^2(1) = 0.071$, $P = 0.790$

Data are depicted as number and percentages [N (%)] of the non-remitter and remitter group based on follow-up data at t1. Chi-square test was applied and two-tailed *P*-values are depicted. $P < 0.05$ was considered statistically significant.

Supplementary Table 6: Interaction and main effects for repeated-measures ANOVAs regarding depression and anxiety scores.

	Interaction effect	Group effect	Time effect
MADRS	$F(2, 111) = 30.442$, $P < 0.001$	$F(2, 111) = 92.151$, $P < 0.001$	$F(1, 111) = 91.107$, $P < 0.001$
HADS-D subscore	$F(2, 111) = 10.336$, $P < 0.001$	$F(2, 111) = 57.747$, $P < 0.001$	$F(1, 111) = 17.296$, $P < 0.001$
HADS-A subscore	$F(2, 111) = 6.097$, $P = 0.003$	$F(2, 111) = 39.163$, $P < 0.001$	$F(1, 111) = 0.072$, $P = 0.789$

Corresponding Supplementary Table to main Table 1. Interaction effect and main effects from repeated-measures ANOVA for the dependent variables MADRS, HADS-D and HADS-A are depicted. $P < 0.05$ was considered statistically significant.

Supplementary Table 7: Group comparisons of depression and anxiety scores.

		never-depressed	non-remitters	remitters	*P*-value non-remitters vs. never-depressed	*P*-value remitters vs. never-depressed	*P*-value remitters vs. non-remitters
MADRS sumscore	t0	3.5±3.6	15.8±7.7	14.7±5.7	<0.001	<0.001	1.000
	t1	0.9±1.4	13.7±9.5	2.6±3.3	<0.001	0.320	<0.001
HADS-D subscore	t0	1.8±1.9	7.4±3.5	6.1±3.4	<0.001	<0.001	0.357
	t1	1.8±1.8	6.5±3.4	2.7±2.4	<0.001	0.353	<0.001
HADS-A subscore	t0	3.3±2.7	8.2±2.2	6.4±3.7	<0.001	<0.001	0.229
	t1	3.6±2.3	10.0±3.9	4.7±3.4	<0.001	0.393	<0.001

Corresponding Supplementary Table to main Table 1. Mean ± standard deviation of clinician-rated (MARDS) and self-reported (HADS-D) depression scores and self-rated anxiety symptoms (HADS-A) are depicted. Baseline (t0) and follow-up (t1) data are shown. Bonferroni-corrected *P*-values relate to changes between groups at indicated timepoint t0 and t1. $P < 0.05$ was considered statistically significant. HADS, Hospital Anxiety and Depression Scale; MADRS, Montgomery-Asperg Depression Rating Scale.

Supplementary Table 8: Interaction and main effects for repeated-measures ANOVAs regarding quality of life scores.

	Interaction effect	Group effect	Time effect
QoL global (%)	$F(2, 111) = 2.881$, $P = 0.060$	$F(2, 111) = 23.673$, $P < 0.001$	$F(1, 111) = 9.323$, $P = 0.003$
QoL physical domain (%)	$F(2, 111) = 3.997$, $P = 0.021$	$F(2, 111) = 26.888$, $P < 0.001$	$F(1, 111) = 20.054$, $P < 0.001$
OoL psychological domain (%)	$F(2, 111) = 7.582$, $P < 0.001$	$F(2, 111) = 46.499$, $P < 0.001$	$F(1, 111) = 7.184$, $P = 0.008$
QoL social domain (%)	$F(2, 111) = 0.121$, $P = 0.886$	$F(2, 111) = 6.170$, $P = 0.003$	$F(1, 111) = 10.342$, $P = 0.002$
QoL environmental domain (%)	$F(2, 110) = 1.830$, $P = 0.165$	$F(2, 110) = 8.810$, $P < 0.001$	$F(1, 110) = 7.194$, $P = 0.008$

Corresponding Supplementary Table to main Table 2. Interaction effect and main effects from repeated-measures ANOVA for the dependent variables global QoL as well as the respective QoL domains measured by WHOQOL-BREF are shown. $P < 0.05$ was considered statistically significant.

Supplementary Table 9: Group comparison of quality of life scores.

		never-depressed	non-remitters	remitters	*P*-value non-remitters vs. never-depressed	*P*-value remitters vs. never-depressed	*P*-value remitters vs. non-remitters
QoL global (%)	t0	74.4±14.1	51.7±15.6	58.3±15.5	<0.001	<0.001	0.574
	t1	76.5±13.7	55.0±17.6	70.1±14.9	<0.001	0.276	0.010
QoL physical domain (%)	t0	81.0±12.6	57.1±16.6	68.8±18.2	<0.001	0.004	0.058
	t1	82.4±13.3	58.1±15.8	80.4±11.6	<0.001	1.000	<0.001
OoL psychological domain (%)	t0	78.0±11.7	51.1±16.8	59.7±14.8	<0.001	<0.001	0.180
	t1	81.4±10.3	53.1±17.6	75.9±13.6	<0.001	0.253	<0.001
QoL social domain (%)	t0	73.7±18.2	58.6±24.8	67.6±16.9	0.017	0.668	0.537
	t1	79.8±14.3	67.2±17.1	74.1±16.6	0.011	0.443	0.585
QoL environmental domain (%)	t0	83.7±9.7	72.7±18.9	75.4±12.5	0.004	0.025	1.000
	t1	85.0±10.3	75.8±8.9	82.6±10.0	0.005	1.000	0.166

Corresponding Supplementary Table to main Table 2. Mean ± standard deviation is depicted. The global score and the respective domains of the World-Health Organization Quality of Life (QoL) questionnaire (WHOQOL-BREF) was used. Baseline (t0) and follow-up (t1) data are shown. Bonferroni-corrected *P*-values relate to changes between groups at indicated timepoint t0 and t1. $P < 0.05$ was considered statistically significant.

Supplementary Table 10: Interaction and main effects for repeated-measures ANOVAs regarding exercise capacity, health behavior, and cardiovascular risk factors.

	Interaction effect	Group effect	Time effect
BMI (kg/m^2)	$F(2, 111) = 1.149$, $P = 0.321$	$F(2, 111) = 7.564$, $P = 0.002$	$F(1, 111) = 14.420$, $P < 0.001$
Drinks/week	$F(2, 111) = 3.255$, $P = 0.042$	$F(2, 111) = 0.899$, $P = 0.410$	$F(1, 111) = 2.366$, $P = 0.127$
Sport	$F(2, 102) = 1.084$, $P = 0.342$	$F(2, 102) = 2.424$, $P = 0.094$	$F(1, 102) = 1.095$, $P = 0.298$
VO_{2max} (ml/kg/min)	$F(2, 92) = 2.580$, $P = 0.081$	$F(2, 92) = 4.414$, $P = 0.015$	$F(1, 92) = 10.713$, $P = 0.001$
WR ind (W/kg)	$F(2, 96) = 2.037$, $P = 0.136$	$F(2, 96) = 5.056$, $P = 0.008$	$F(1, 96) = 1.581$, $P = 0.212$
LVEF (%)	$F(2, 109) = 2.191$, $P = 0.117$	$F(2, 109) = 0.102$ $P = 0.903$	$F(1, 109) = 1.688$, $P = 0.197$
LVEDD (mm)	$F(2, 110) = 0.623$, $P = 0.538$	$F(2, 110) = 2.103$, $P = 0.127$	$F(1, 110) = 0.017$, $P = 0.896$
NT-proBNP (ng/l)	$F(2, 108) = 0.613$, $P = 0.544$	$F(2, 108) = 0.153$, $P = 0.859$	$F(1, 108) = 3.629$, $P = 0.059$
CRP (mg/l)	$F(2, 109) = 0.495$, $P = 0.611$	$F(2, 109) = 2.038$, $P = 0.135$	$F(1, 109) = 4.847$, $P = 0.030$
NYHA class	$F(2, 107) = 0.874$, $P = 0.420$	$F(2, 107) = 1.333$, $P = 0.268$	$F(1, 107) = 2.264$, $P = 0.135$

Corresponding Supplementary Table to main Table 3. Interaction effect and main effects from repeated-measures ANOVA for indicated dependent variables are depicted. $P < 0.05$ was considered statistically significant. BMI, body mass index; CRP, C-reactive protein; LVEDD, left ventricular end-diastolic parameter; LVEF, left ventricular ejection fraction; NT-proBNP, N-terminal prohormone of brain natriuretic peptide; NYHA, New-York Heart Association Classification; VO_{2max}, maximum oxygen uptake; WR ind maximum work rate indexed to bodyweight.

Supplementary Table 11. Group comparisons of exercise capacity, health behavior, and cardiovascular risk factors.

		never-depressed	non-remitters	remitters	*P*-value non-remitters vs. never-depressed	*P*-value remitters vs. never-depressed	*P*-value remitter vs. non-remitters
BMI (kg/m²)	t0	24.1±3.7	27.7±4.8	24.1±2.6	0.002	1.000	0.016
	t1	24.8±4.1	29.2±5.3	24.7±3.0	<0.001	1.000	0.006
Drinks/week	t0	2.2±3.5	0.6±1.1	0.7±1.2	0.165	0.166	1.000
	t1	1.8±2.5	1.1±1.5	3.0±9.8	1.000	0.961	0.657
Sport	t0	3.64±1.56	2.92±1.38	3.03±1.50	0.364	0.366	1.000
	t1	3.71±1.41	2.85±1.34	3.65±1.62	0.147	1.000	0.402
VO_{2max} (ml/kg/min)	t0	28.3±7.6	23.7±6.4	23.2±6.5	0.175	0.066	1.000
	t1	25.5±7.0	19.9±6.1	23.3±5.0	0.015	0.829	0.391
WR ind (W/kg)	t0	2.16±0.60	1.74±0.56	1.86±0.64	0.062	0.250	1.000
	t1	2.11±0.56	1.56±0.49	1.93±0.50	0.002	0.735	0.213
LVEF (%)	t0	56.7±8.7	59.0±8.3	59.1±11.6	1.000	0.997	1.000
	t1	57.7±7.9	56.8±8.9	56.9±8.7	1.000	1.000	1.000
LVEDD (mm)	t0	54.5±7.7	55.1±5.2	50.9±4.8	1.000	0.160	0.263
	t1	54.5±6.1	54.2±5.6	52.0±3.6	1.000	0.278	0.832
NT-proBNP (ng/l)	t0	207±296	202±261	161±184	1.000	1.000	1.000
	t1	221±266	263±210	208±237	1.000	1.000	1.000
CRP (mg/l)	t0	3.5±6.6	6.1±9.1	2.5±3.5	0.494	1.000	0.373
	t1	2.4±3.3	3.2±2.2	1.3±0.9	1.000	0.372	0.167
NYHA class	t0	1.23±0.54	1.40±0.63	1.33±0.59	0.882	1.000	1.000
	t1	1.29±0.56	1.60±0.74	1.33±0.59	0.186	1.000	0.598

Corresponding Supplementary Table to main Table 3. Continuous variables are depicted as mean ± standard deviation and categorical variables are shown as percentages as indicated. Baseline (t0) and follow-up (t1) data are depicted. Bonferroni-corrected *P*-values relate to changes between t0 and t1 within the respective groups. $P < 0.05$ was considered statistically significant. BMI, body mass index; CRP, C-reactive protein; LVEDD, left ventricular end-diastolic parameter; LVEF, left ventricular ejection fraction; NT-proBNP, N-terminal prohormone of brain natriuretic peptide; NYHA, New-York Heart Association Classification; VO_{2max}, maximum oxygen uptake; WR ind maximum work rate indexed to bodyweight.
